# Analysis of LhcSR3, a Protein Essential for Feedback De-Excitation in the Green Alga *Chlamydomonas reinhardtii*

**DOI:** 10.1371/journal.pbio.1000577

**Published:** 2011-01-18

**Authors:** Giulia Bonente, Matteo Ballottari, Thuy B. Truong, Tomas Morosinotto, Tae K. Ahn, Graham R. Fleming, Krishna K. Niyogi, Roberto Bassi

**Affiliations:** 1 Dipartimento di Biotecnologie, Università di Verona, Verona, Italy; 2 Department of Plant and Microbial Biology, University of California, Berkeley, California, United States of America; 3 Physical Biosciences Division, Lawrence Berkeley National Laboratory, Berkeley, California, United States of America; 4 Dipartimento di Biologia, Università di Padova, Padova, Italy; 5 Department of Chemistry, University of California, Berkeley, California, United States of America; Kyoto University, Japan

## Abstract

In photosynthetic organisms, feedback dissipation of excess absorbed light energy balances harvesting of light with metabolic energy consumption. This mechanism prevents photodamage caused by reactive oxygen species produced by the reaction of chlorophyll (Chl) triplet states with O_2_. Plants have been found to perform the heat dissipation in specific proteins, binding Chls and carotenoids (Cars), that belong to the Lhc family, while triggering of the process is performed by the PsbS subunit, needed for lumenal pH detection. PsbS is not found in algae, suggesting important differences in energy-dependent quenching (qE) machinery. Consistent with this suggestion, a different Lhc-like gene product, called LhcSR3 (formerly known as LI818) has been found to be essential for qE in *Chlamydomonas reinhardtii*. In this work, we report the production of two recombinant LhcSR isoforms from *C. reinhardtii* and their biochemical and spectroscopic characterization. We found the following: (i) LhcSR isoforms are Chl a/b– and xanthophyll-binding proteins, contrary to higher plant PsbS; (ii) the LhcSR3 isoform, accumulating in high light, is a strong quencher of Chl excited states, exhibiting a very fast fluorescence decay, with lifetimes below 100 ps, capable of dissipating excitation energy from neighbor antenna proteins; (iii) the LhcSR3 isoform is highly active in the transient formation of Car radical cation, a species proposed to act as a quencher in the heat dissipation process. Remarkably, the radical cation signal is detected at wavelengths corresponding to the Car lutein, rather than to zeaxanthin, implying that the latter, predominant in plants, is not essential; (iv) LhcSR3 is responsive to low pH, the trigger of non-photochemical quenching, since it binds the non-photochemical quenching inhibitor dicyclohexylcarbodiimide, and increases its energy dissipation properties upon acidification. This is the first report of an isolated Lhc protein constitutively active in energy dissipation in its purified form, opening the way to detailed molecular analysis. Owing to its protonatable residues and constitutive excitation energy dissipation, this protein appears to merge both pH-sensing and energy-quenching functions, accomplished respectively by PsbS and monomeric Lhcb proteins in plants.

## Abstract 2

To prevent photodamage by excess light, plants use different proteins to sense pH changes and to dissipate excited energy states. In green microalgae, however, the LhcSR3 gene product is able to perform both pH sensing and energy quenching functions.

## Author Summary

Reactive oxygen species are formed during photosynthesis, particularly when electron transport is saturated in high light. The process of non-photochemical quenching (NPQ) helps protect plants against excess light by dissipating the excited states of chlorophyll into heat. By doing so, it prevents the formation of triplet excites that otherwise would react with molecular oxygen to form singlet oxygen, a damaging reactive oxygen species. In plants, NPQ is triggered by the PsbS protein, which senses pH changes caused by excess light and consequently triggers energy-quenching functions in other proteins. The green microalga *C. reinhardtii* lacks the PsbS proteins, and NPQ depends on the LhcSR3 protein. In this study, we show that, unlike PsbS, LhcSR3 not only binds pigments but is also a strong quencher for chlorophyll excited states. LhcSR3 carries protonatable residues that enable it to sense pH change. Its quenching activity is further enhanced by low pH, suggesting that this algal protein merges the functions of pH sensor and of excited state quencher into a single gene product.

## Introduction

In photosynthetic organisms, feedback dissipation of chlorophyll (Chl) singlet excited states balances light harvesting with metabolic energy consumption, in order to prevent photodamage due to reactive oxygen species (ROS) formation when excess energy is transferred to O_2_. Both plants and algae can dissipate Chl excited states into heat through mechanisms involving xanthophyll-binding Lhc proteins.

The light-harvesting complex (Lhc) gene family is present in all photosynthetic eukaryotes [1]. Lhc proteins act in light harvesting, owing to their capacity to bind Chl and carotenoid (Car) chromophores, a characteristic shared by most members of the family, with few exceptions [2]. Lhc proteins are also involved in photoprotection through their xanthophyll ligands, which are active in quenching Chl singlets and triplets as well as in scavenging ROS [3]–[9], with lutein and zeaxanthin (Zea) playing a predominant role [10],[11].

Among Lhc proteins, LhcSR, PsbS, and ELIPs are more specifically involved in photoprotective mechanisms and are over-expressed under stress [12]–[14]. ELIPs, transiently expressed in plants [15], repress Chl biosynthesis by sequestering precursors in order to prevent free Chl accumulation in high light (HL) [16]. PsbS acts in chloroplast lumenal pH sensing [17],[18] and in activation of the fast component energy-dependent quenching (qE) of non-photochemical quenching (NPQ) [17],[19]. Photosynthetic organisms thermally dissipate light energy absorbed in excess with respect to their needs for photosynthesis through NPQ. LhcSR orthologs are widely distributed among green and brown algae, and are also found in some mosses [20],[21]. Knock-out mutants disrupted in *psbS* and *lhcSR3.1/lhcSR3.2* genes have similar qE-null phenotypes, respectively, in plants and algae [17],[22], suggesting similar functions and mechanisms of action for their gene products. Biochemical analysis of PsbS, both in vivo and in vitro, identified two lumenal-exposed, dicyclohexylcarbodiimide (DCCD)–binding glutamate residues essential for qE triggering [18],[23],[24]. PsbS has also been shown to be unable to bind pigments, owing to the non-conservation of Chl-binding residues [19],[25]. Thus, protonation of PsbS leads to activation of a lutein- and Zea-dependent quenching process in Lhcb proteins [19],[26]. Consistent with this model, deletion of PSII antenna subunits affects qE kinetics and amplitude [27]–[30], and these subunits have been shown in vitro to be active in energy-quenching processes involving the formation of Zea and/or lutein radical cations, by means of a Chl-Car charge-transfer quenching (CT quenching) mechanism [6],[31],[32].

Although the biogenesis of LhcSR proteins has been studied [14], their biochemical properties are still unknown. For information on the mechanism by which LhcSR activates energy dissipation in *C. reinhardtii*, we characterized LhcSR3 isoforms after in vitro refolding of the purified apoprotein, a procedure that has been shown to be effective in yielding pigment-protein complexes with the same biochemical and spectral properties as many Lhc proteins [6],[32]–[35]. We show that LhcSR3, unlike PsbS, forms complexes with pigments containing Chl a, Chl b, lutein, and violaxanthin/Zea. Spectroscopic analysis of LhcSR3 shows the presence of very short fluorescence lifetimes compared with other members of the Lhc family, implying that energy-dissipating mechanisms are very active in this protein. These findings, together with the capacity of LhcSR3 to bind DCCD, a marker for proton-sensitive residues in proteins, and its increased quenching activity upon acidification, suggest that it combines the functions of pH sensor and of the Chl excited state quenching needed for NPQ, which in plants is performed by two distinct protein components: PsbS and Lhcb subunits.

## Results

### LhcSR Accumulation and Post-Translational Modification

**Figure 1:**
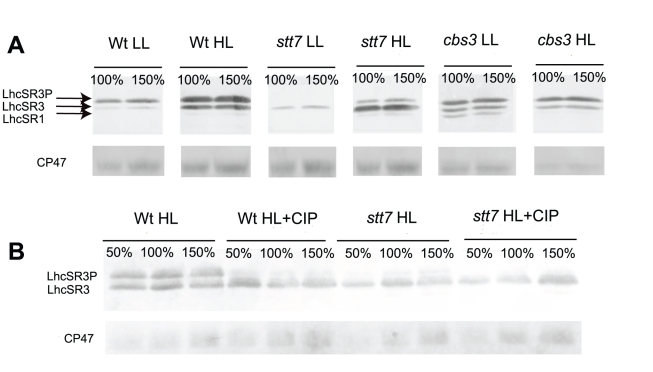
Immunoblotting detection of LhcSR and CP47 on *C. reinhardtii* thylakoids with specific antibodies. (A) LhcSR on thylakoids from WT, *stt7* mutant, and *cbs3* mutant cells, HL and LL acclimated. Two sample dilutions are loaded (100%  = 0.8 µg of Chl). Arrows indicate three cross-reacting bands; the lowest is poorly represented. (B) LhcSR on WT and *stt7* HL thylakoids untreated or treated with calf intestine alkaline phosphatase (CIP). Three sample dilutions were loaded (100%  = 0.8 µg of Chl).

**Figure 2:**
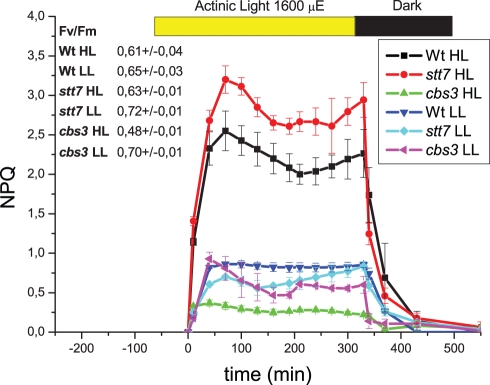
NPQ analysis of *C. reinhardtii* cells (WT, *stt7,* and *cbs3* genotypes), HL or LL long-acclimated. Fv/Fm for each sample shown in left inset.

In *C. reinhardtii*, LhcSR3 has been reported to be essential for qE, consistent with its increased abundance in thylakoid membranes upon acclimation of cells at HL, a condition which up-regulates qE capacity [22]. In *C. reinhardtii,* three genes (*lhcSR1, lhcSR3.1,* and *lhcSR3.2*) encode LhcSR isoforms [36], but two of them, *lhcSR3.1* and *lhcSR3.2*, encode the same 259-amino-acid polypeptide. The LhcSR1 isoform has 253 residues with 87% identity with respect to LhcSR3.1/LhcSR3.2. For information on the properties of the LhcSR proteins, we cloned and expressed LhcSR1 and LhcSR3 (corresponding to genes *lhcSR1* and *lhcSR3.1*) in *Escherichia coli*. The LhcSR3 isoform, purified from inclusion bodies, was injected into rabbits to obtain an antiserum that was found to recognize both the LhcSR1 and LhcSR3 recombinant proteins in SDS-PAGE (data not shown). Figure 1 shows the immunodetection of LhcSR following SDS-PAGE separation of thylakoid membranes from HL (500 µE) and low light (LL) (50 µE) acclimated cells: three bands are detected at approximately 25 kDa. The fastest migrating band was always faint, whereas the bands with higher apparent molecular weight (MW) were strongly over-accumulated in HL matching results from a recent report [22]. To check whether the retardation of Lhc protein bands in SDS-PAGE was the result of protein phosphorylation, as previously shown for CP29 [37], we treated samples with alkaline phosphatase (Figure 1B). The intensity of the upper band in the HL lane was considerably decreased by the treatment, with concomitant intensification of the intermediate band. Similar effects of phosphatase treatment were observed in the LL sample, suggesting that phosphorylation of LhcSR3 did not depend on light intensity. In order to verify the possibility that LhcSR3 phosphorylation is involved in NPQ, we proceeded in two steps. We first verified that phosphorylation was almost absent in the *stt7* mutant, which lacks the thylakoid kinase responsible for LHCII phosphorylation and State 1–State 2 transitions [38],[39]. We then compared the NPQ kinetics of wild type (WT) and *stt7* upon acclimation to LL and HL conditions. Figure 2 shows that NPQ amplitude was below 0.5 in WT LL cells, but increased to 2.5 in HL acclimated cells. NPQ in *stt7* HL cells was even higher than in WT. These results imply that Stt7 kinase is the major agent responsible for phosphorylation of LhcSR3, and that phosphorylation is not needed for NPQ activity.

Most Lhc proteins bind both Chl a and Chl b, but LhcSR proteins are also found in organisms, like diatoms, that lack Chl b [20]. We therefore analyzed the Chl b–less *cbs3* mutant [40] in order to test whether this pigment species was required for LhcSR stability in vivo. Figure 1 demonstrates that this is not the case, since the same three bands were present in both mutant and WT. However, upon HL acclimation, the two upper LhcSR3 bands did not increase as in WT. Consistent with the similar level of LhcSR3 accumulation, NPQ was similar to WT in LL cells (Figure 2).

Titration of LhcSR3 protein abundance in thylakoid membranes can be performed by exploiting the availability of a specific antibody. For this purpose, various dilutions of thylakoid membranes from HL-grown cells were loaded on an SDS-PAGE gel, together with a dilution series of the recombinant pigment-protein obtained as described below. Following transfer to nitrocellulose and immunodetection with anti-LhcSR-specific antibody, the intensity of the immunological reaction was estimated by densitometry and related to the amount of Chl loaded. Based on a PSII/PSI ratio of 1.18 and antenna sizes of 240 and 222 Chls/reaction Center for PSI and PSII, respectively [41], and on the proposed number of Chls bound per LhcSR3 polypeptide of 6.7±1.9 (see below), we calculated a LhcSR/PSII ratio of 0.17±0.11 in HL acclimated thylakoids. Although this estimation should be used with caution, it clearly suggests that LhcSR is substoichiometric with respect to PSII reaction centers.

### Aggregation State of LhcSR

**Figure 3:**
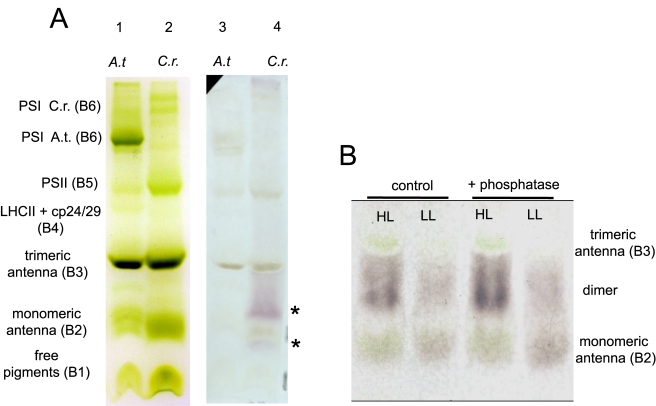
Native Deriphat-PAGE and Western blotting. (A) Native Deriphat-PAGE electrophoresis and Western blotting of HL *C. reinhardtii* (*C.r.*) cell membranes showing anti-LhcSR immuno-reacting bands (asterisks) at monomeric antenna protein MW and between monomeric and trimeric antennae MW. Note how, in native electrophoresis, band resolution is always much worse than in SDS-PAGE electrophoresis, and background signal is higher. *A. thaliana* (*A.t.*) membranes are for comparison. (B) Native Deriphat-PAGE separation of both HL and LL native complexes and Western blotting with anti-LhcSR, showing how phosphatase treatment does not influence LhcSR aggregation state.

In order to clarify the function of LhcSR3, it is essential to establish its possible interactions with other proteins. We therefore separated pigment-protein complexes after solubilization with α-dodecyl-maltoside by native electrophoresis, as shown in Figure 3. As previously shown [42], the band with the highest mobility, at the electrophoretic front, contains protein-free pigments, and the slower migrating bands represent pigment-protein complexes or their oligomers (Figure 3). When thylakoids from HL- and LL-grown cells were separated and immunoblotted with the anti-LhcSR antibody, two reactive bands were detected, migrating, respectively, to the level of green band 2 and between green bands 2 and 3. Since LhcSR apoproteins have a MW similar to that of the monomeric Lhc proteins forming band 2, this indicates that LhcSR in thylakoid membranes form dimers in both HL and LL conditions. In principle, LhcSR may also form heterodimers with a similar MW protein, e.g., a monomeric Lhcb subunit, such as CP29, CP26, and/or Lhcbm1 [43]. In order to check this possibility, we proceeded to a second-dimension separation of native gel lanes in denaturing conditions, followed by immunoblotting with antibodies specific for Lhcb proteins [44]. No co-migration of Lhcb proteins or LhcSR corresponding to the upper LhcSR-reactive band was detected, thus excluding stable interactions between LhcSR and Lhcb proteins (data not shown). Alkaline phosphatase treatment did not affect this pattern (Figure 3B), indicating that phosphorylation plays no role in dimer formation.

### Pigments Binding to LhcSR1 and LhcSR3

**Figure 4:**
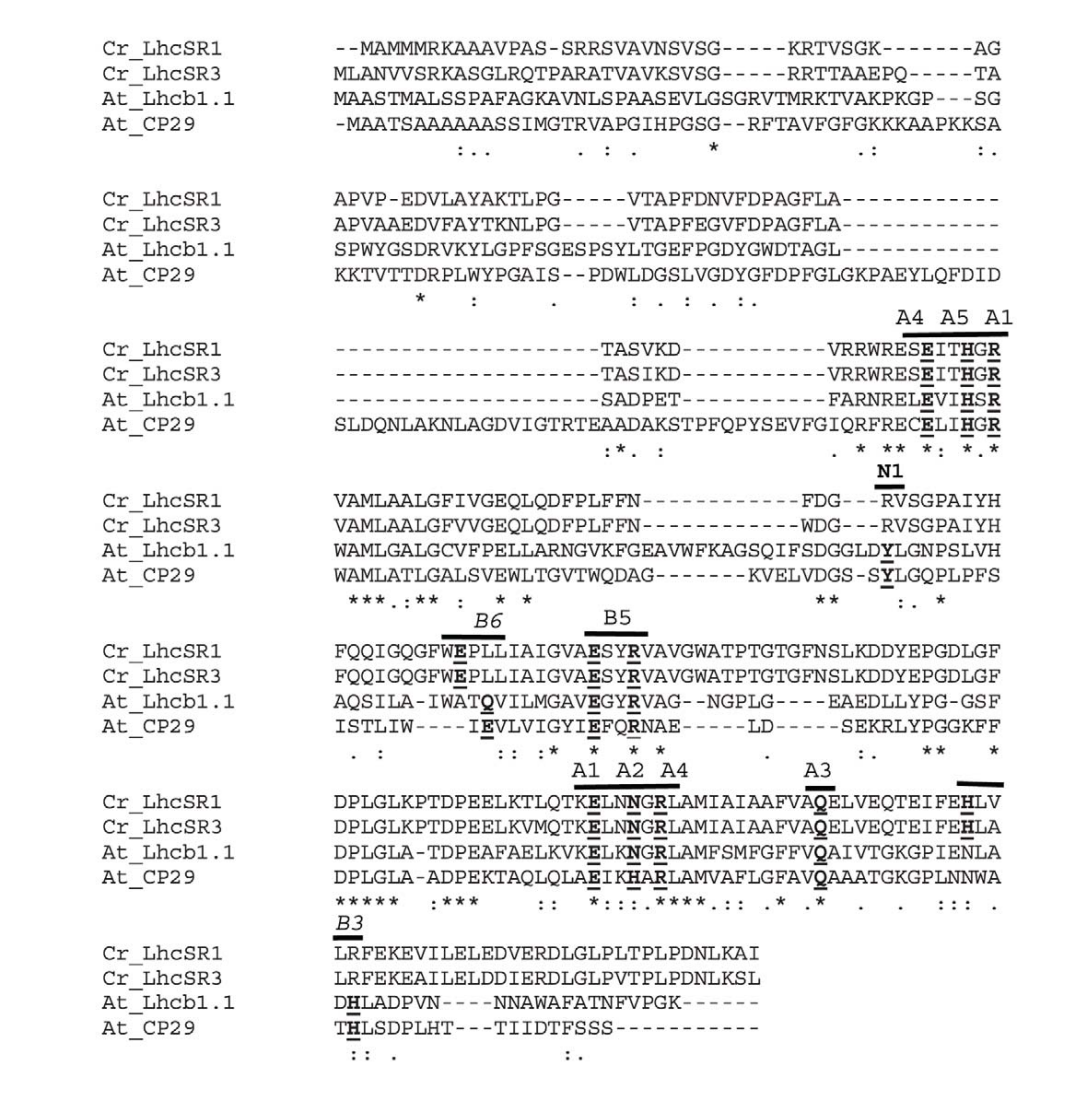
Sequence alignment of LhcSR1, LhcSR3, and *A. thaliana* CP29 and Lhcb1. Chl- and neoxanthin-coordinating residues are shown. Note conservation of all these residues in both LhcSR isoforms, with exception of residues coordinating B3 and B6, with respect to CP29 and Lhcb1. At, *A. thaliana*; Cr, *C. reinhardtii*.

In order to clarify the role of a protein in energy dissipation, it is essential to assess its capacity for binding pigments. For example, PsbS, essential for NPQ in plants [17], was suggested to be the actual quenching site, but its role was later revised after it was found not to bind pigments [19],[25]. The sequence alignment of LhcSR versus two *Arabidopsis thaliana* Lhcb sequences (Lhcb1 and CP29) in Figure 4 shows that six out of eight amino acid residues responsible for Chl binding in LHCII and CP29 [33],[35],[45],[46] are conserved, i.e., residues coordinating four Chl a–specific sites (A1, A2, A4, and A5) and two Chl a/Chl b promiscuous sites (A3 and B5). The two other residues (B3 and B6 sites) are not conserved. Sequence requirements for xanthophyll-binding residues are not well known, with the exception of tyrosine residue 111 (147) in Lhcb1 (CP29), which is involved in neoxanthin binding [47]: this tyrosine is not conserved in LhcSR1 or LhcSR3, indicating that no neoxanthin binding takes place.

In order to demonstrate pigment-binding capacity, the protein can be purified from *C. reinhardtii* cells acclimated to HL. Unfortunately, this approach was not successful, because of the presence of many other Lhc subunits with similar physico-chemical properties, hindering isolation of this low-abundance subunit. An alternative approach, successfully used in similar circumstances, consists of over-expressing the encoding gene in bacteria and refolding the apoprotein in vitro with pigments, giving rise to a holoprotein with biochemical and spectroscopic properties identical to those of the purified protein, as demonstrated in a large variety of Lhc members [2],[33],[34],[48]–[51].

The in vitro reconstitution of recombinant LhcSR1 and LhcSR3 yielded a pigment-binding complex having the same mobility as monomeric Lhcb proteins in a sucrose gradient. Refolding efficiency was higher in LhcSR3 than in LhcSR1. Since the yield of isoform LhcSR1 was limiting for full biochemical and spectroscopic characterization, we focused attention on the LhcSR3 isoform only, which is more physiologically important, as the subunit has been shown to be essential for NPQ.

**Figure 5:**
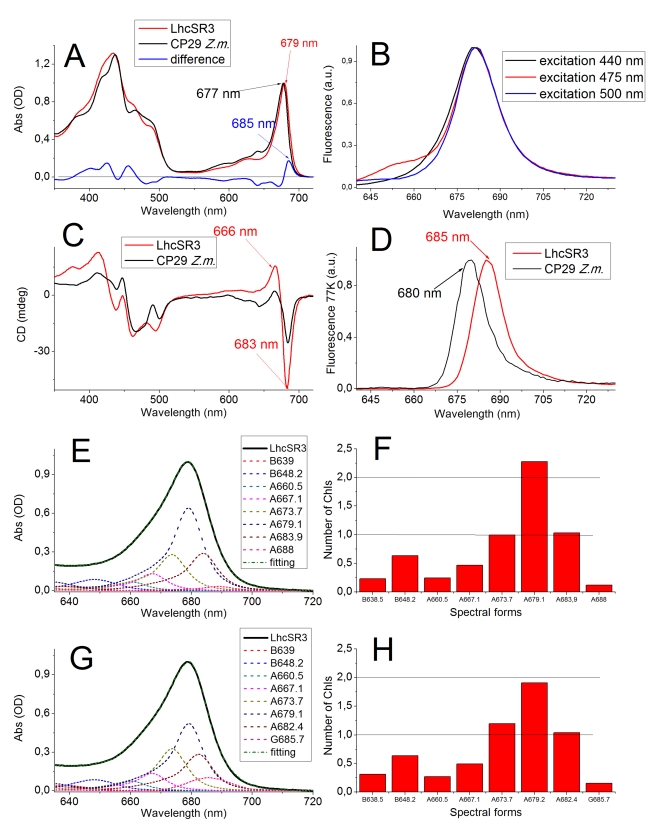
Spectroscopic analysis of LhcSR3. (A) Absorption spectra in visible region of LhcSR3 (red line) and CP29 (*Z. mays,* black line); maximum absorption peak in Qy region is also shown. (B) LhcSR3 fluorescence emission at 300K with excitation at 440 nm (black line, Chl a), 475 nm (red line, Chl b), and 500 nm (blue line, Cars). (C) CD spectra in visible region of LhcSR3 (red line) and CP29 (*Z. mays,* black line); Chl a (666 and 683 nm) major peaks are shown. (D) Fluorescence emission spectra at 77K (630 to 720 nm) of LhcSR3 (red line) and CP29 (*Z. mays,* black line) upon excitation of Chl a (440 nm); peak values are shown. (E) Deconvolution of LhcSR3 absorption spectrum in Qy spectral region with Chl spectral forms in protein environment. Several spectral forms are marked A (Chl a) and B (Chl b), with peak absorptions reported in the box insert. (F) Number of Chls represented by spectral forms in (E): data are normalized for six total Chls. (G) Deconvolution of LhcSR3 absorption spectrum in Qy spectral region with Chl spectral forms in protein environment and a single Gaussian with 16 half-height bandwidths peaking at 685.7 nm; spectral forms are marked A (Chl a), B (Chl b), and G (Gaussian function). (H) Number of Chls represented by spectral forms of (G): data are normalized for six total Chls.

Figure 5A shows the LhcSR3 absorption spectrum with a Qy transition peak at 678.8 nm, i.e., strongly shifted with respect to the 670-nm peak of the free Chl a in detergent solution, and even more red-shifted than any PSII antenna protein so far analyzed. The fluorescence emission spectra (Figure 5B) were characterized by a 681-nm peak, independently of exciting Chl a (440 nm), Chl b (475 nm), or xanthophylls (495 nm), implying efficient energy transfer between pigments, although a low level of direct emission from uncoupled Chl b was detected. These data, all together, show that LhcSR3 forms stable complexes with Chl a, Chl b, and xanthophylls, with chromophores having mutual interactions similar to those previously described for Lhcb proteins.

Quantitative analysis of chromophore binding to LhcSR3 was carried out by determination of Chl binding to the complex versus dye binding to apoprotein [52]. With the well-characterized Lhcb1 recombinant protein, binding 12.6 Chl per polypeptide [35],[53], as a reference, a Chl/apoprotein stoichiometry of 6.7±1.9 was obtained for LhcSR3. On the basis of this stoichiometry and on the conservation of six out of eight Chl-binding residues, we propose a stoichiometry of six Chls per apoprotein (Table 1), which is the lowest figure ever calculated for an Lhc complex, even below the eight Chl per CP29 holoprotein [54]. Nevertheless, a figure of seven Chl per polypeptide cannot be excluded. High-performance liquid chromatography (HPLC) analysis (Table 1) showed that LhcSR3 is characterized by a high Chl a/b ratio (Chl a/b  = 6.3±0.3), demonstrating strong affinity for Chl a. We also performed in vitro reconstitution with Chl a and xanthophylls only, in the absence of Chl b, and again obtained a pigment-protein complex with characteristics similar to those of the control (reconstituted in the presence of Chl a and Chl b), including a red-shifted absorption peak at 678.4 nm and efficient excitation energy transfer from xanthophyll to Chl a. In that complex, Chl b chromophores were substituted by Chl a, as shown by the decreased absorption at 635–645 nm accompanied by an increased absorption at 660–675 nm in the difference absorption spectra (Figure S1). Xanthophyll composition was the same as in the sample containing Chl b (Table 1), thus confirming that LhcSR3 does fold well in the absence of Chl b. It is worth noting that there was a 20-fold decrease in refolding efficiency with respect to the Chl b–containing sample, indicating that Chl b, although not indispensable, does contribute to pigment-protein complex stability.

**Table 1:** Pigment content in picomoles of refolded LhcSR3 complexes in absence or presence of Zea (LV and LVZ, respectively) and with or without Chl b.

Refolded Complex	Chls (a + b) Normalization	Pigment Content							
		Chl a	Chl b	Chl/Car	Cars	Violaxanthin	Lutein	Zea	Chl a/b Ratio
LV LhcSR3	100.0	86.3	13.7	2.5	39.8	18.0	21.4	0.0	6.3
LVZ LhcSR3	100.0	83.5	16.5	2.6	38.3	14.9	14.6	8.8	5.1
LhcSR3 without Chl b	100.0	100.0	0.0	2.9	35.0	14.2	20.7	0.0	—
LV LhcSR3	6.0	5.2	0.8	2.5	2.4	1.1	1.3	0.0	6.3
LVZ LhcSR3	6.0	5.0	1.0	2.6	2.3	0.9	0.9	0.5	5.1
LhcSR3 without Chl b	6.0	6.0	0.0	2.9	2.1	0.9	1.2	0.0	—
LV LhcSR3	7.0	6.0	1.0	2.5	2.8	1.3	1.5	0.0	6.3
LVZ LhcSR3	7.0	5.8	1.2	2.6	2.7	1.1	1.0	0.6	5.1
LhcSR3 without Chl b	7.0	7.0	0.0	2.9	2.4	1.0	1.5	0.0	—
*A.thaliana* CP29	8.0	6.0	2.0	4.1	2.0	0.5	1.0	0.5	3.1
*A.thaliana* Lhcb1	12.0	7.0	5.0	3.8	3.1	0.3	2.0	0.8	1.4

Data are averages of five independent experiments, standard deviation <10%. *A. thaliana* CP29 and Lhcb1 pigment contents are reported for comparison (data from [6] and [31]).

### Car Binding Site Occupancy in the Presence of Violaxanthin and/or Zea

Besides Chl a and Chl b, Lhc proteins bind xanthophylls into specific sites, i.e., sites L1, L2, N1, and V1 [45]–[47],[55],[56]. However, in various members of the Lhc family, the affinity of each site for xanthophyll species is variable, and confers functional specialization on different Lhc proteins.

HPLC analysis of reconstituted LhcSR3 shows lutein and violaxanthin bound to the apoprotein, whereas neither neoxanthin nor loroxanthin, although present in the pigment mix during refolding, were bound. Based on six Chls per polypeptide and a Chl/Car ratio of 2.5, the number of Cars per apoprotein molecule in LhcSR3 is between two and three, indicating the presence of two binding sites with strong selectivity for lutein and violaxanthin, probably sites L1 and L2. A third binding site, partially unoccupied, is also present. The same conclusions hold, even under the assumption of seven Chls per polypeptide.

The absence of neoxanthin in the refolded complex indicates that site N1 is absent, consistent with the non-conservation of tyrosine 121, as in the case of CP24 [47]. The third Car binding site is thus of V1 type. When LhcSR3 was reconstituted in the presence of Zea (LhcSR3 LVZ), the Chl/Car and Chl a/b ratios were the same and slightly decreased, respectively, relative to the control protein. Since the transition energies of xanthophylls are tuned differentially by binding to different protein sites [55],[57], spectral deconvolution of the absorption spectra in the Soret region was performed as in [57]–[60] on the LV and LVZ complexes, in order to identify the Zea-binding sites. Results are shown in Figure S2. In all solutions yielding the best fits, the xanthophyll absorption forms showed three levels of red shifts with respect to absorption in organic solvent, i.e., 18–19, 15–16, and 9 nm, consistent with L2, L1, and V1 binding sites, respectively [55]. In the presence of Zea (0.5 mol per mole of protein), multiple new absorption forms were needed for optimal fitting, implying that Zea entered the three sites, although to different extents. This was unexpected, since in other recombinant Lhc proteins Zea binds selectively to site L2, and a single spectral form is needed for fitting [60].

### Light Absorption Properties of LhcSR3

Information on chromophore organization within pigment-proteins can be obtained by circular dichroism (CD) [61]. The LhcSR3 CD spectrum (Figure 5C) has features previously reported for native and recombinant Lhcb proteins, with signals in the Qy region at 683 nm (−) and 660 nm (+). In the Soret range, a strong broad negative signal is observed at 495 nm, associated with xanthophylls [61]. Interestingly, the amplitude of the CD signal in the Qy region is almost twice as strong as that of the homologous protein CP29 from *Zea mays*, upon normalization at the same protein molar concentration, which indicates an enhanced level of excitonic interactions between Chl a chromophores in LhcSR3 [61].

The absorption spectrum of LhcSR3 is shown in Figure 5A, compared with that of CP29 from *Z. mays* [33], used as a reference, since it has the most similar pigment-binding properties among all known Lhc proteins. The Qy transition of LhcSR3 peaks at 679 nm, 2 nm red-shifted compared with CP29, and has a tail that is redder than in CP29. Analysis of this Chl spectral contribution to the LhcSR3 LV absorption spectrum with Chl a and Chl b spectral forms in a protein environment (Figure 5E and 5F) yielded six major spectral forms, including four Chl a forms, peaking at 667, 674, 679, and 684 nm (accounting for 0.5, 1.0, 2.3, and 1.2 Chls, respectively). Chl b spectral forms were detected at 639 and 648 nm, together accounting for nearly one Chl b molecule per polypeptide. Two additional low-amplitude absorption forms (0.15× Chl a), peaking at 660 nm and 688 nm, were indispensable for optimal fitting of the spectra. Similar analysis in CP29 or any other Lhcb polypeptide did not require such strongly red-shifted spectral forms. The transition energy of absorption forms is quite well conserved within the Lhcb family, and the red-most component peaks at 682 nm [5],[33],[35]. In LhcSR3, the red-most forms were further red-shifted to 684 and 688 nm. Such strong shifts have been reported in the PSI-associated LHCI proteins to be caused by excitonic interactions and accompanied by increased bandwidths [49],[62], indicating that the 684- and 688-nm forms represent a single, wider absorption form deriving from excitonic interaction of two Chl a molecules. This possibility was probed by performing deconvolution, including a Gaussian spectral component with larger bandwidth in the red-most part of the spectrum (Figure 5G and 5H), and the goodness-of-fit was in fact significantly improved. The best description of the experimental spectrum was obtained by using a Gaussian peaking at 685.7 nm, with 16 nm full width at half maximum, compared with 12 nm of a monomeric Chl a spectral form [57]; the 684- and 688-nm forms disappeared. The spectrum of Zea-binding LhcSR3 was also best fitted with a 16-nm spectral form peaking at 685.7 nm, implying that no major conformational changes were induced by binding of Zea (data not shown).

The presence of low-energy excited states in LhcSR3 was confirmed by the 77K fluorescence emission peak at 685 nm, substantially red-shifted compared with CP29 (680 nm) (Figure 5D).

### Time-Resolved Fluorescence Analysis

**Figure 6:**
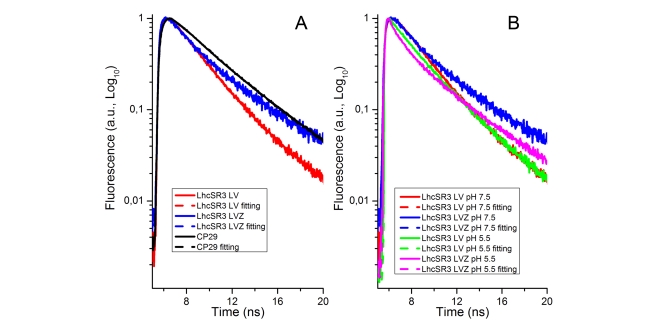
Time-resolved fluorescence analysis. Fluorescence emission kinetics of CP29 and LhcSR3 lutein/violaxanthin (LhcSR3 LV) or lutein/violaxanthin/Zea (LhcSR3 LVZ) binding was recorded at 685 nm (A). Fluorescence emission kinetics of LhcSR3 lutein/violaxanthin (LhcSR3 LV) or lutein/violaxanthin/Zea (LhcSR3 LVZ) was recorded at pH 7.5 or pH 5.5 (B). Decay curves were fitted with two or three exponential functions for CP29 and LhcSR3. Fitted curves are shown with dashed lines.

Red-shifted spectral forms have been involved in both energy dissipation [6],[29],[63] and light harvesting [49]. We therefore proceeded to single photon counting fluorescence lifetime analysis (Figure 6) in order to verify whether LhcSR3 acts as a quencher of the antenna proteins, which are well known to have a long lifetime, on the order of 3–4 ns [64],[65]. Decay curves upon excitation at 435 nm and detection at 685 nm are shown in Figure 6, and the results of their deconvolution are listed in Table 2. As a reference, we also analyzed a sample of CP29. Matching previous studies, CP29 showed two lifetimes of 4.6 ns (62%) and 1.8 ns (38%), thus yielding an average lifetime of 3.5 ns [66]. The case of LhcSR3 was clearly different, since three exponential components were needed for best fitting of the decay curves. Besides two components of 4 and 1.95 ns, accounting, respectively, for 10% and 25% of total decay, a dominant short component was obtained, with a lifetime less than 100 ps, accounting for nearly 65% of fluorescence. Similar results were obtained when fluorescence emission was collected at different wavelengths. LhcSR3 binding Zea (LhcSR3 LVZ) also showed decay with three components, like LhcSR3 LV. However, the intermediate lifetime component was slightly faster, 1.5 ns versus 1.95 ns, and the longest component had increased amplitude, probably because of partial uncoupling of a small fraction of pigment (Figure 6; Table 2). When measurements were repeated at pH 5.5, fluorescence decays were even faster because of increased amplitude of the fastest component, a shortening of the intermediate component (τ2), and decreased amplitude of the long-living component (τ3). In this case the Zea-containing sample has a shorter intermediate-lifetime component at 1.1 ns and a longer τ3 component. These effects, together, result in an average lifetime very similar to that of the LV sample. It is worth mentioning that no difference in fluorescence decay was observed in CP29 upon acidification.

**Table 2:** Lifetimes and relative fluorescence quantum yields of CP29 and LhcSR3.

Protein (pH)	A1	τ1	A2	τ2	A3	τ3	τ Average
CP29 (pH 7.5)	n.d.	n.d.	37.5%	1.8	62.5%	4.6	3.5
CP29 (pH 5.5)	n.d.	n.d.	34.6%	1.8	65.4%	4.6	3.7
LhcSR3 LV (pH 7.5)	65.3%	<0.1	24.7%	2.0	10.0%	4.1	0.9
LhcSR3 LVZ (pH 7.5)	60.9%	<0.1	23.0%	1.5	16.0%	5.2	1.2
LhcSR3 LV (pH 5.5)	76.8%	<0.1	19.9%	1.3	3.3%	3.3	0.4
LhcSR3 LVZ (pH 5.5)	69.9%	<0.1	25.3%	1.1	4.8%	4.7	0.5

Lifetimes (ns) (τ1–τ3) and relative amplitudes (A1–A3) of exponential components are shown, together with average lifetime of each sample (τaverage).

n.d., not detected.

### Near-Infrared Transient Absorption Kinetics and Spectra in LhcSR3

A recently proposed mechanism for qE involves the transient formation of a Chl^−^Car^+^ radical cation, followed by charge recombination to the ground state (CT quenching). The formation of Car radical cation species can be detected by near-infrared (NIR) transient absorption (TA) spectroscopy, both in intact systems [67] and in purified Lhcb proteins [6],[31].

**Figure 7:**
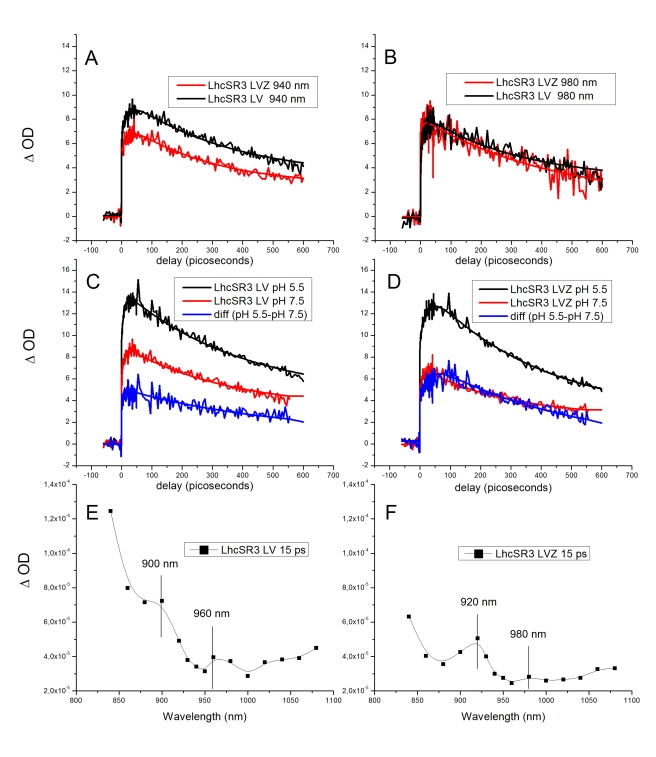
TA in the NIR. (A and B) TA traces from LhcSR3 LV and LhcSR3 LVZ at 940 nm (A) and 980 nm (B). (C and D) TA traces at 940 nm from LhcSR3 LV (C) and LhcSR3 LVZ (D) at pH 7.5 versus pH 5.5. (E and F) LhcSR3 LV (E) and LhcSR3 LVZ (F) TA spectra with fixed 15-ps time.

We performed ultrafast TA measurements on LhcSR3 samples by exciting Chl a at 670 nm and recording the absorption decay in the picoseconds timescale at 980 and 940 nm (NIR), respectively corresponding to Zea and lutein radical cations [31],[68]. In the NIR spectral region, Chl-excited state absorption was detected in addition to Car radical species. However, Chl-excited state absorption can easily be distinguished according to its kinetics, characterized by decay components only, whereas Car^+^ also displays a rise component. Figure 7 shows TA traces from LhcSR LV and LVZ samples. At both 980- and 940-nm detections, both LV and LVZ traces display a clear rise component, followed by a decay similar to what was previously described for higher plant monomeric Lhc proteins involved in NPQ [6],[67]. In the present study, both quenching and Car cation TA signal were a constitutive property of the LhcSR3 protein. Thus, the kinetic parameters were obtained directly from decay curves [31] rather than from quenched-minus-unquenched difference kinetic curves [6]. At 940 nm, the rise time was 14±3 ps, and decay time 428±103 ps, in both samples. The rise time at 980 nm was 7±4 ps, and decay time 501±224 ps. Interestingly, unlike previous results obtained in Lhc complexes from plants, the amplitude of the fast-rising component at 980 nm was not enhanced in the LVZ sample relative to the LV sample, indicating that the contribution of Zea radical cations to Zea-binding LhcSR3 is not dominant. Consistently, the LV sample displayed a clear rise component at both wavelengths, with similar kinetics. This indicates that Zea binding is not essential for Car radical cation formation in LhcSR3, at variance with previous findings in higher-plant monomeric Lhcb proteins [6],[31]. We then proceeded to verify the effect of pH on the formation of Car radical cation(s). To this aim we repeated the measurements upon lowering the pH to 5.5. Results (Figure 7C and 7D) show that the amplitude of the TA signal at 940 nm is increased by 40% and 90%, respectively, in LV and LVZ samples upon acidification. At 940 nm, the fastest component (<1 ps) is enhanced in both complexes at pH 5.5, while an increased amplitude of the slow rise component (∼23 ps) was observed only in LhcSR LVZ. It was previously reported that both the fastest and slowest rise components of TA are associated to Car radical cation formation, the former being related to energy transfer among Chls strongly coupled with the Chl-Car heterodimer responsible for charge separation, and the latter being associated with energy transfer among Chls in the complex [69]. This effect was not observed at 980 nm, consistent with the minor contribution of Zea in the process.

For information on which xanthophyll species are involved in generating the TA signal of LhcSR3, we reconstructed the NIR TA spectrum by recording kinetic traces in the 840–1,080 nm region and plotting TA signals at a delay time of 15 ps, which corresponds to peak amplitude. The resulting spectra are shown in Figure 7E and 7F. Major contributions are observed at various wavelengths, i.e., 920 and 980 nm in the LVZ sample, with the peaks of shorter wavelengths exhibiting the highest amplitude. The LV spectrum was shifted by 20 nm (900 and 960 nm). The signal amplitude at shorter wavelengths rose again towards 850 nm, the spectral range where violaxanthin radical cations are expected [68]. Although the signal-to-noise ratio of our NIR TA data decreases below 900 nm, violaxanthin involvement in CT quenching cannot be excluded in LhcSR3. Thus, LhcSR3 appears to have a high yield of radical cation(s) and also the capacity to produce this chemical species from various xanthophylls in the presence or absence of Zea. This is at variance with plant Lhcb proteins, in which Zea contributes to CT quenching both directly [6],[31] and as an allosteric activator of lutein CT quenching [32].

### Effect of Protonation and Zea on NPQ In Vivo

**Figure 8:**
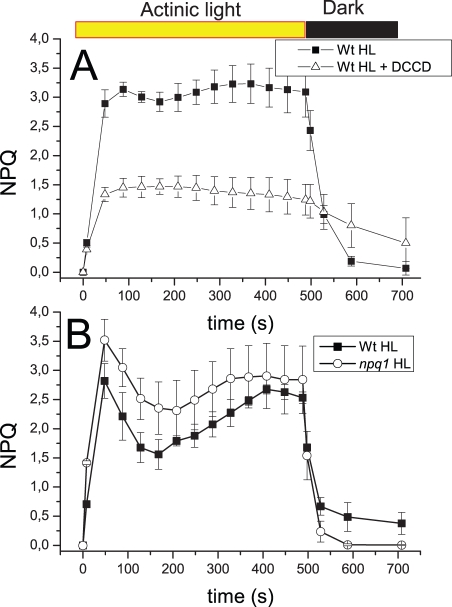
In vivo effect of DCCD and Zea on NPQ. (A) measurement of NPQ in HL acclimated *C. reinhardtii* cells with or without incubation with 20 µM DCCD. (B) NPQ analysis of *C. reinhardtii* cells (WT and *npq1* genotypes) HL long acclimated (500 µmol m^−2^ s^−1^). Note that WT strain used in the experiment shown in (B) is CC425 for comparison with *npq1*mutant in the same genetic background [70]; WT in (A) is *cw15*.

The above data (Figures 6 and 7) clearly suggest that acidification up-regulates quenching in LhcSR3, while Zea binding has a smaller effect. In order to further assess whether the effects observed in vitro on the isolated LhcSR3 protein are reflected in the level and/or kinetics of NPQ in vivo, we proceeded in two steps. First, we verified that NPQ in *C. reinhardtii* is sensitive to DCCD, a protein-modifying agent specific for reversibly protonatable residues. NPQ kinetics of HL acclimated cells with and without incubation with 20 µM DCCD is shown in Figure 8A and clearly demonstrates a strong inhibitory effect of DCCD on NPQ in vivo. In the second step, we measured NPQ kinetics in the *npq1* mutant [70], unable to synthesize Zea, relative to in WT. The total NPQ amplitude was similar in the two strains. If anything, it was somehow rising faster and higher in the Zea-less mutant. Also, the dark recovery was faster in the *npq1* mutant. We verified that the HL treatment was effective in inducing Zea synthesis in WT but not in the *npq1* mutant by analyzing pigment content by HPLC before and after the actinic light treatment (1,600 µmol m^−2^ s^−1^). In doing that, we observed that HL-grown WT cells did contain a significant amount of Zea even after the 1-h dark incubation before the onset of the illumination, and the de-epoxidation index increased from 0.2 to 0.4 during measurement (Table S1). It should be noted that this behavior is significantly different from the case of higher plants, in which Zea is absent in dark-adapted plants, and the de-epoxidation index reaches 0.6 upon HL exposure.

### DCCD Binding to LhcSR3, as Compared with Other Lhcb Proteins

**Figure 9:**
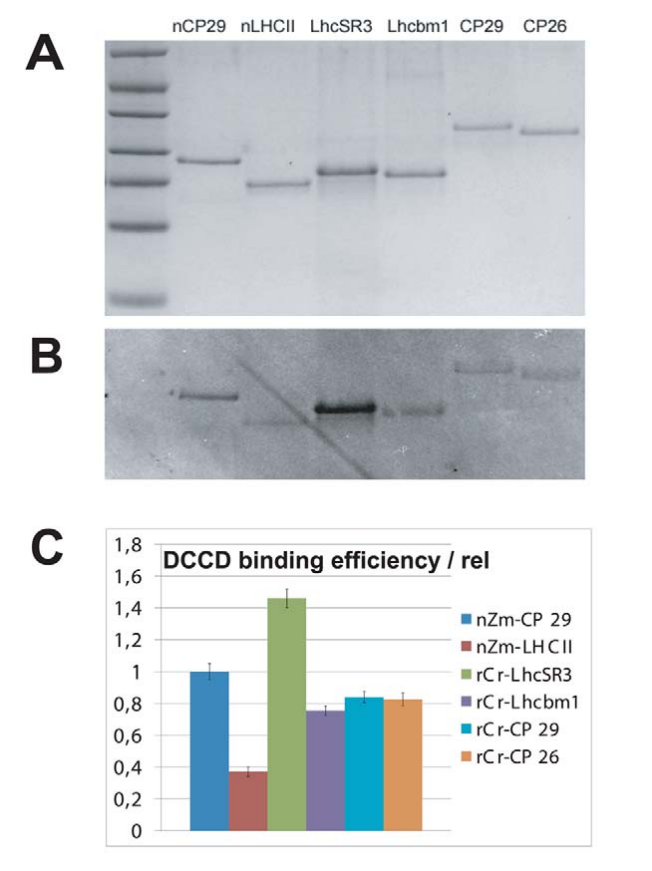
DCCD-labeling experiment. (A) SDS-PAGE separation of proteins labeled with ^14^C-DCCD. (B) Autoradiography of gel shown in (A). (C) Densitometric analysis of bands in (B) after normalization to Coomassie staining (A).

Feedback energy dissipation is triggered by low lumenal pH. In plants, pH transduction is operated by PsbS through the protonation of two glutamate residues, which can be identified by labeling with DCCD, a chemical covalently binding to protonatable protein sites [18]. Although a *psbS* gene is present, the PsbS protein is not accumulated in algae [71], thus opening the question as to whether LhcSR is the molecule responsible for pH-dependent triggering of qE in algae. This hypothesis is supported by the presence of several acidic residues, potential candidates as pH-sensors in the LhcSR lumenal region sequence. We verified the capacity of LhcSR to bind DCCD by labeling the recombinant protein with ^14^C-DCCD, followed by autoradiography. Other Lhcb proteins, including CP29 from plants carrying a DCCD-binding site [72] and the algal CP29, CP26, and Lhcbm1, were analyzed for comparison. The results are shown in Figure 9: LhcSR3 revealed very efficient binding of DCCD, higher than plant CP29. It is interesting to note that algal Lhcb proteins show DCCD binding of approximately 50% with respect to LhcSR3, but clearly higher than plant LHCII, indicating that some level of pH responsiveness may be a general property of algal PSII antenna proteins (Figure 9B and 9C).

## Discussion

A recent report showed that although a *psbS* gene is present in algae, the corresponding protein is not accumulated [71]. This is of particular interest, since the *npq4* mutant in *C. reinhardtii*, blocked in thermal dissipation, is disrupted in the *lhcSR3.1* and *lhcR3.2* genes encoding identical Lhc-like proteins [14],[20],[22]. This implies that the mechanism of feedback de-excitation differs in algae versus plants and that PsbS action can be carried out by a different component(s). The properties of LhcSR3 are thus of primary importance for understanding qE in algae.

### Expression, Phosphorylation, and Aggregation State of LhcSR3

Accumulation of LhcSR3 strongly depends on light intensity during growth: of the three LhcSR immuno-reactive bands that can be resolved by SDS-PAGE, the upper two are strongly up-regulated in HL-grown cells, whereas the fast-migrating isoform is of low intensity and decreases in HL. The correspondence of these bands with the three *lhcSR* genes found in the *C. reinhardtii* genome [36] is based on the fact that mutants deleted in the *lhcSR3.1* and *lhcSR3.2* genes are also missing the two higher bands [22], consistent with predictions based on polypeptide MW, with *lhcSR1* encoding a smaller protein than *lhcSR3.1* and *lhcSR3.2*. LhcSR1 and LhcSR3 are both over-expressed in HL [22] and iron starvation conditions [73]. The two bands with higher apparent MW correspond to the phosphorylated and unphosphorylated LhcSR3 isoforms, according to the results of phosphatase treatment (Figure 1). The increase in LhcSR3 accumulation strongly correlates with the amplitude of NPQ, consistent with the report that LhcSR3 protein is responsible for high NPQ levels in *C. reinhardtii* [22]. We show here that the *stt7* mutant [39] is unable to phosphorylate LhcSR3 to any significant extent, yet it exhibits NPQ as in WT, or even higher. We thus conclude that phosphorylation is not indispensable for NPQ, but interpret the increased NPQ in *stt7* as a consequence of the block in State 1–State 2 transitions, a mechanism active in energy pressure balancing in algae [74], thus increasing PSII over-excitation and the need for energy dissipation through NPQ. Phosphorylation does not appear to affect the aggregation state of LhcSR either, as detected by native Deriphat-PAGE (Figure 3). Migration of dimeric Lhc proteins between monomeric and trimeric Lhcbs has been reported for Lhca1–Lhca4 [2],[42],[75]. Since LhcSR1 is much less abundant than LhcSR3, and as we found no evidence for the presence of other Lhcb proteins in dimers, homodimeric organization is most likely, although alternative hypotheses cannot be entirely excluded.

### LhcSRs Are Pigment-Binding Proteins

Pigment binding is an important property for evaluating the role of LhcSR in excitation energy quenching: a pigment-binding protein may be directly involved in the quenching reaction [6], whereas a non-pigment-binding protein cannot, although it may play an ancillary role such as pH sensing, as previously found for PsbS [18],[19]. Both LhcSR isoforms are here shown to form stable and specific complexes with Chl and xanthophyll chromophores, as clearly demonstrated by (i) the spectral shift induced by pigment-protein interactions (Figure 5), (ii) the capacity for excitation energy transfer from Chl b and xanthophylls to Chl a, and (iii) for the LhcSR3 isoform, the strong optical activity: free pigments in detergent solution or unspecifically bound to proteins have very low amplitude CD spectra, with a single broad positive component in the Qy region [34]. Thus, recombinant LhcSR has the same properties as Lhc antenna complexes, unlike PsbS, which cannot form pigment proteins in vitro or in vivo [19],[25]. It is worth noting that, since LhcSR can coordinate pigments in vitro, it is highly unlikely that this does not occur in vivo. Although nonspecific binding of Chl to proteins cannot, in principle, be excluded, it is hard to imagine that coordination is carried out in such a specific way that it provides stoichiometric binding of Chls and xanthophylls, CD signals, and efficient energy transfer between chromophores, essentially very similar to other members of Lhc protein family, without reflecting an original capacity for pigment binding in vivo. The conservation of six pigment-binding residues with respect to other Lhc protein members further supports the pigment-binding nature of LhcSR3.

As regards the number and organization of chromophores in LhcSR3, the Chl/protein ratio indicates six or seven Chls per polypeptide, consistent with the non-conservation of Chl-binding residues at B3 and B6 binding sites compared with CP29, which binds eight Chl [33] (Figure 4). Interestingly, the Chl b complement is slightly below one per polypeptide: while Chl b may have a stabilizing effect because of the establishment of hydrogen bonds through its vinyl group [46], a small fraction of the LhcSR3 pigment-protein complexes may bind only Chl a and xanthophylls. This is consistent with the observation that LhcSR3 protein accumulates in the *cbs3* mutant, lacking Chl b, and the finding of LhcSR orthologs in diatoms, which lack Chl b [76]. In fact, a Chl a/lutein/violaxanthin complex may be obtained with spectral properties and pigment composition similar to the Chl b–containing holoprotein.

**Figure 10:**
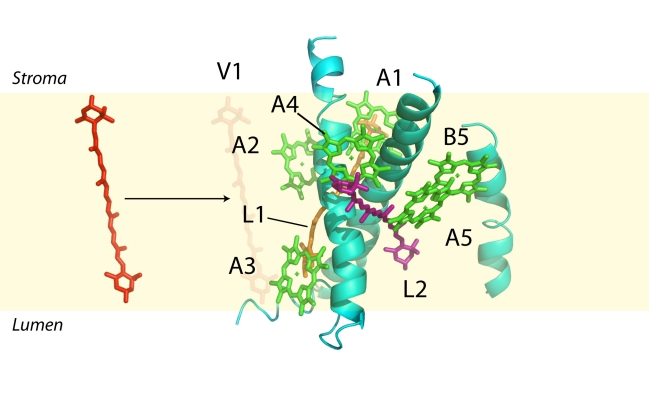
Model showing Chl and xanthophyll chromophores bound to different sites in LhcSR3. Model was built by homology based on crystal structure of LHCII by [46].

Lutein and violaxanthin are the major xanthophylls in LhcSR3; neoxanthin and loroxanthin are absent. Neoxanthin is mainly bound to the major LHCII trimeric antenna [77],[78] and is involved in scavenging superoxide anions, not in qE [3]. The function of loroxanthin, still unknown, is probably related to enhancing light-harvesting efficiency, since its content is increased in LL cells and decreased in HL conditions [78]. Based on six Chls per polypeptide, more than two xanthophylls per polypeptide are calculated with lutein and violaxanthin bound, respectively, to sites L1 and L2, although site selectivity is less strict than in the case of most Lhc proteins [56],[58],[60],[79]–[81], not only for lutein and violaxanthin but also for Zea. According to its spectral shift (Figure S2), the additional xanthophyll ligand is bound to a third V1-like site. A model of the LhcSR3 holoprotein with bound pigments is shown in Figure 10.

Although the recombinant proteins obtained by in vitro reconstitution are monomers, as determined from their mobility in sucrose gradient and native PAGE (data not shown), the native state of the complex appears to be at least partly dimeric (Figure 3). So far, dimeric Lhcs have been reported for PSI only, i.e., Lhca1/Lhca4 and Lhca2/Lhca3 [2],[82]. We cannot exclude the possibility that LhcSR dimers have other properties in addition to those reported here, due to pigment-pigment interactions between subunits and/or additional pigments bound at protein-protein interfaces [52],[83]. Nevertheless, recombinant Lhca proteins have been shown to have all the major biochemical and functional properties typical of LHCI complexes isolated from thylakoids [49].

### LhcSR3 Involvement in Energy Dissipation

Accumulation of LhcSR3 is greatly enhanced in HL conditions (Figure 1) [22] or nutrient deficiency [73]. This is similar to the expression pattern of other Lhc-like gene products such as PsbS and ELIPs, whose involvement in photoprotection mechanisms has been well documented [15],[16],[18] and contrasts with the case of the major LHCII antenna complex, whose expression/accumulation is enhanced in light-limiting conditions [12],[84]. Also, like PsbS [85] and ELIPs, LhcSR3 is present in substoichiometric amounts with respect to the PSII reaction center. These characteristics indicate that it is involved in excitation energy dissipation in order to prevent photoinhibition, and that its activity/abundance can be modulated, depending on environmental requirements. The substoichiometric amount of a strong quenching molecule does not exclude the possibility of its being an efficient quencher for photosystems II with a high degree of connectivity [86]. Even though we have evidence that LhcSR3 might fulfill a PsbS-like role as a sensor of lumen acidification, as supported by its decrease in lifetime (Figure 6; Table 2) and by the presence of protonatable DCCD-binding residues (Figure 9), our data suggest that it displays intrinsic capacity for direct excitation energy quenching. This conclusion is supported by the pigment-binding capacity and the spectroscopic properties of LhcSR3: the dominant fluorescence lifetime components, shorter than 100 ps, imply that an energy dissipation channel is constitutively active in recombinant LhcSR3, and its activity is further enhanced upon acidification. Other Lhc proteins have much slower fluorescence decay rates, in the range of 3.4–4.5 ns, consistent with their function as sensitizers for photosystem II [65]. Faster components (1.4–2.8 ns) have been resolved in low abundance, deriving from alternative conformations [64]. In the case of LhcSR3, the dominant lifetime is below 100 ps, suggesting that a third state of the protein is stabilized, having strong energy dissipation activity. Nevertheless, a significant fraction of the fluorescence was detected with a lifetime of 1.95 ns. Since energy equilibration within a monomeric Lhc protein is completed within a few picoseconds [87]–[89], we suggest that the two lifetime components (<100 ps versus 1.95 ns) are due to different molecular forms whose abundance is regulated by pH. Heterogeneity may be related to the lower-than-one stoichiometry of Chl b versus LhcSR apoprotein, implying that a particular site may be occupied by either Chl a or Chl b. One additional source of heterogeneity is evidenced by the resolution of two Chl b spectral forms (Figure 5) deriving from binding to distinct protein sites, matching previous reports on Lhc proteins [33],[90]. According to the hypothesis that Chl a–Chl a excitonic interactions lead to red-shifting of the LhcSR3 spectrum (Figure 5), occupancy by Chl b of a site potentially involved in interactions would prevent excitonic coupling, because of the large difference in site energy of the two chromophores. In CP29, Chl a in binding site B5 can undergo excitonic interaction with Chl a in site A5, thus promoting CT quenching [6]. We propose that excitonic interaction between Chl a molecules in sites A5 and B5 is involved in the quenching process active in LhcSR3, and that the heterogeneity of site B5 occupancy is responsible for lifetime heterogeneity. This matches the fact that Chl A5 is a Chl a–specific site in all Lhc proteins, whereas site B5 is promiscuous in monomeric Lhcs [33],[90]. Spectral/lifetime heterogeneity may (also) be provided by mixed Chl a/b occupancy of site A3 [35].

Energy dissipation in CP29 has been reported to derive from the transient formation of a Zea radical cation and a Chl anion, followed by charge recombination to the ground state [67] in an Lhcb protein domain including Chl A5, Chl B5, and Car in site L2 [6]. Triggering of the energy dissipation reaction is obtained by displacement of violaxanthin in site L2 with Zea or lutein: this event induces a conformational change, leading to the establishment of an excitonic interaction between Chl a molecules in sites A5 and B5 [6],[32]. The coupled Chl dimer is more favorable to CT quenching, because the charge delocalization over the two Chls lowers the energy requirement for CT quenching [6]. We observed a 684–688 nm red-shifted absorption form in LhcSR3 with large bandwidth, which is not present in CP29 or in any other Lhcb protein so far described, to our knowledge. We suggest that this form derives from the strong excitonic interaction between Chl A5 and B5, constitutively present in this protein, without the need for binding of Zea. This view is supported by the high levels of Car radical cations measured in LhcSR3 (Figure 7), approximately ten times higher than in plant Lhcb4–Lhcb6 proteins in their active, Zea-binding form. With the concomitant presence of the short fluorescence lifetime component (<100 ps), we conclude that LhcSR3 is predominantly stabilized in the energy dissipation conformation that is transiently induced in plant monomeric Lhcb4–Lhcb6 proteins. Mutation analysis is in progress in order to confirm this hypothesis. To our knowledge, this is the first example of an Lhcb protein exhibiting a dominant dissipative conformation when isolated in detergent solution. Besides CT quenching, direct energy transfer from a Chl a Qy transition to a lutein S1 state [91] or a Chl-Chl charge transfer [92] have been proposed as alternative mechanisms for qE. Although we have no evidence that these processes are important for energy dissipation in algae, we cannot exclude the possibility that they may contribute to quenching. In fact, the fast lifetime component we resolved in LhcSR3 is below 100 ps, i.e., it is significantly faster than the relaxation of the lutein radical cation (see Results), indicating the involvement of multiple quenching mechanisms. More detailed spectroscopic analysis is needed to assess whether CT quenching is the only component of qE in algae or whether other mechanisms are also involved, as well as high-resolution structural studies on LhcSR3 in order to elucidate the molecular architecture of the quenching site in its active, energy-dissipating state.

### Different qE-Triggering Mechanisms in Plants and Algae?

Energy quenching is dependent on lumenal pH in both plants and algae, as clearly shown by its sensitivity to uncouplers [93] and the inhibition of NPQ by DCCD (Figure 8A). Yet, algae lack PsbS, the sensor of lumenal pH [18],[19]. The observation that the quenched conformation of the LhcSR3 protein is stable in detergent solution (i.e., in the absence of a transmembrane pH gradient) raises the question of how qE is modulated by the onset of light: in fact, active quenching in the dark or in LL conditions would impair photosynthesis and cell growth. In order to explain pH regulation of quenching in algae, we propose that LhcSR3, although present as an active quencher in thylakoids, is disconnected from other Lhc proteins, thus minimizing energy dissipation, while it establishes interactions with PSII antenna component(s) upon lumen acidification and protonation of lumen-exposed, negatively charged residues both in LhcSR and in PSII antenna components. This model is consistent with both earlier and new observations. First, lack of Lhcbm1, a major component of trimeric LHCII, has been shown to reduce qE strongly [43]. Second, the *cbs3* mutant, although accumulating LhcSR3 in HL, cannot develop high NPQ, perhaps because of the lack of an Lhcbm1 partner for LhcSR3. Low lumenal pH also increases the formation of lutein radical cation (Figure 7C and 7D) and increases the amplitude of short-living fluorescence lifetime components (Figure 6B) in the isolated protein, suggesting that lumen acidification, besides promoting connection of the LhcSR quencher to the light-harvesting antenna system, also enhances the quenching activity of the pigment-protein complex.

Besides low lumenal pH, an additional factor in triggering NPQ in plants is synthesis of Zea in excess light. Zea is incorporated into Lhc proteins [60],[94],[95] and promotes dissociation of a pentameric Lhc complex, which is needed to trigger NPQ [27]. The fluorescence lifetime of isolated LhcSR3 in detergent solution is not strongly affected by Zea. The *npq1* mutation, preventing Zea synthesis, has been shown to decrease NPQ in plants [10], while in *C. reinhardtii* the effect of Zea is much reduced [26],[95]. We observed essentially the same NPQ activity in WT and in the *npq1* mutant, consistent with the small effect of Zea on the lifetime properties of LhcSR3 in vitro and with LhcSR3 being essential for NPQ in vivo [22]. It is thus possible that some level of NPQ dependence on Zea can be observed in some conditions as a consequence of its binding to antenna protein interacting with LhcSR3, possibly Lhcbm1 [43].

### Conclusions

We have shown that LhcSR3, essential for energy quenching in *C. reinhardtii,* is a pigment-binding protein with the properties of a constitutive quencher, since it has a short lifetime component (<100 ps) when isolated in detergent solution. This is different from the case of plant monomeric Lhcb proteins, which have long lifetimes and whose quenching mechanisms are activated in vivo by the action of the PsbS protein and/or Zea synthesis. We propose that LhcSR3 regulates energy dissipation by establishing reversible interactions with other Lhcb antenna proteins, in particular Lhcbm1 [43], and that these interactions are induced by low lumenal pH through protonatable DCCD-binding sites present in both Lhcb proteins and LhcSR3. Thus, LhcSR3 has the properties of both an energy quencher, a function catalyzed by Lhcb proteins in vascular plants [6],[31],[91], and a sensor for lumenal pH, which is a function of PsbS in plants [18],[19],[27].

## Materials and Methods

### Cell Growth Conditions

WT strains *cw15* [96] and CC425 (*Chlamydomonas* Genetics Center, Duke University) and mutants *stt7* [97], *npq1* [70], and *cbs3* [40] *C. reinhardtii* cells were grown in high-salt medium [96] at light regimes of 500 and 50 µmol m^−2^ s^−1^ for HL and LL samples, respectively. In Figure 8B, the *npq1* mutant and WT were from the CC425 strain [70].

### NPQ Measurements

Cells acclimated to HL or LL conditions were harvested in the exponential growth phase (∼2×10^6^ cells/ml), pelleted, and resuspended at a concentration of ∼10^8^ cells/ml). Cells were pre-illuminated for 2 min with a weak (3 µmol m^−2^ s^−1^) far-red LED before NPQ analysis with a PAM-101 (Waltz); actinic light was 1,600 µmol m^−2^ s^−1^ and saturating light, 4,080 µmol m^−2^ s^−1^. The far-red LED was kept on during dark recovery. In the experiment of Figure 8A, cells were pre-incubated with 20 µM DCCD (Sigma) for 15 min in the dark before measurements.

### Membrane Preparation

*C. reinhardtii* thylakoids were purified as previously described [98], and membrane dephosphorylation was carried out by incubating one sample at 28°C for 1 h in the presence of calf intestinal alkaline phosphatase (1 Unit/3 µg Chl).

### SDS-PAGE Electrophoresis and Immunoblotting

Denaturing SDS-PAGE as described previously [52] was performed in the presence of 6 M Urea with the Tris-sulfate acrylamide and Tris-glycine buffer systems [99]. The gel was transblotted to a nitrocellulose filter, decorated with an anti-LhcSR serum, and developed by means of the alkaline phosphatase detection system.

### Native Electrophoresis

Thylakoid membranes were solubilized in the presence of 1.2% α-dodecyl-maltoside and loaded on native electrophoresis gels [42].

### Molecular Cloning and Apoprotein Expression

The DNA sequence coding mature LhcSR1 and LhcSR3.1 was cloned in the pET28 vector (Novagen) and transformed in *E. coli* BL21de3 cells. The recombinant proteins were purified as inclusion bodies from bacterial lysate as previously described [34].

### In Vitro Reconstitution of Pigment-Apoprotein Complex

The refolding procedure was performed as described in [34].

### Spectroscopy

CD spectra were obtained with a Jasco J-600 spectropolarimeter with scan rate 200 nm/min. Absorption spectra were obtained with an AMINCO DW2000 spectrophotometer, with scan rate 2 nm/s, bandwidth 1 nm, and optical path length 1 cm. Fluorescence spectra were obtained at room temperature with a Fluoromax 3 fluorometer (Horiba Jobin Yvon). Time-resolved fluorescence spectroscopy was carried out at room temperature with the single-photon-timing method on a FluoTime 200 from PicoQuant. Kinetics were analyzed with FluoFit from PicoQuant. Excitation was at 435 nm, and detection was at 680, 690, 700, and 710 nm.

### Pigment Analysis

Pigments were extracted from pelleted cells, and samples were frozen in liquid nitrogen and resuspended in 80% acetone buffered with Na_2_CO_3_. The supernatant of each sample was then recovered after centrifugation (15 min at 15,000 *g*, 4°C). Separation and quantification of pigments was performed by HPLC [100]. Chl a/b and Chl/Car ratios were corrected through fitting analysis of the absorption spectrum [80].

### NIR TA Measurements

The NIR TA laser system has previously been described [6],[31],[67]. Briefly, the repetition rate was 250 kHz, and the pump pulses were tuned to ∼670 nm. The maximum pump energy and full width at half maximum of the pulse auto-correlation trace were ∼24 nJ/pulse and ∼40 fs, respectively. White light continuum probe pulses were generated in a 1-mm quartz plate. Observation of the cross-correlation function of the pump and probe overlap was approximately 85 fs. The mutual polarizations of the pump and probe beams were set to the magic angle (54.7°). A monochromator (Spectra Pro 300i; Acton Research) and an InGaAs photodiode (DET410; Thorlabs) were used to monitor transmission. A sample cell for isolated LHCs with a path length of 1 mm was chilled by a circulating water bath (VWR Scientific 1160; PolyScientific) set at 7°C during data acquisition to prevent sample degradation. For TA measurements at lower pH, samples were placed in 40 mM citrate buffer (pH 5.5) with 0.2% α-dodecyl-maltoside.

### DCCD Binding

Recombinant *C. reinhardtii* Lhcbm1, CP29, and CP26 were expressed in *E. coli* and refolded with pigments [34]. *Z. mays* LHCII and CP29 were purified in their native form as described in [72]. All samples were labeled with ^14^C-DCCD (Amersham) following the methods of [72] and loaded on SDS-PAGE electrophoresis gels [101]. After Coomassie staining, gels were dried, and radioactivity was revealed through autoradiography.

## Supporting Information

Figure S1 **Absorption spectrum of LhcSR3 refolded in the presence of Chl a only.** Sample is compared with LhcSR LV. Refolding yield in presence of Chl a is only 25- to 30-fold lower than in presence of both Chl a and Chl b. Spectra are normalized to number of Chls bound per apoprotein.
(0.06 MB EPS)

Figure S2 **Deconvolution of absorption spectra of LhcSR3 in Soret region.** Spectral deconvolution of LhcSR3 reconstituted in absence (LhcSR LV) (A) or presence (LhcSR LZV) (B) of Zea. Absorption spectra were analyzed in terms of contribution of individual pigments with absorption spectra of pigments in LHC proteins. Since multiple solutions were possible, only solutions consistent with biochemical pigment composition, such as Chl a/b, Chl/Car ratios, and Car content, were chosen. Among remaining possible solutions, those with lowest discrepancy with original absorption spectra were chosen. Fitting procedure was performed in both cases with three Chl a (Chl a 1, Chl a 2, and Chl a 3; red traces), two Chl b (Chl b 1 and Chl b 2; blue traces), and the spectral form in protein environment properly shifted in/to Soret region. For LhcSR LV, five additional Car spectral forms were applied for best fitting: two luteins (Lut 1 and Lut 2; pink traces), shifted by 16 and 19 nm compared with absorption in organic solvent, two violaxanthins (Vio 1 and Vio 2; dash-dot traces), shifted by 15 and 18 nm, and a fifth generic Car spectral form (obtained by averaging lutein and violaxanthin spectral forms), shifted by only 9 nm. In the LhcSR LVZ sample, similar Car spectral forms were applied, minus the Lut 2 spectral form, which was not necessary, and with the addition of one Zea spectral form (Zea 2; brown trace), shifted by 19 nm compared with Zea absorption in organic solvent.
(0.15 MB EPS)

Table S1 **Picomoles of Zea, violaxanthin, and antheraxanthin before and after NPQ induction.** De-epoxidation Index (Dep. Index) is calculated as (*Z* + *A*/2)/(*Z* + *V* + *A*). Data about *A. thaliana* from [102].
(0.02 MB PDF)

## Abbreviations

Car: carotenoid
CD: circular dichroism
Chl: chlorophyll
CT quenching: charge-transfer quenching
DCCD: dicyclohexylcarbodiimide
HL: high light
HPLC: high-performance liquid chromatography
LL: low light
MW: molecular weight
NIR: near-infrared
NPQ: non-photochemical quenching
qE: energy-dependent quenching
ROS: reactive oxygen species
TA: transient absorption
WT: wild type
Zea: zeaxanthin
