# Supplementary material for: Analysis of LhcSR3, a Protein Essential for Feedback De-Excitation in the Green Alga Chlamydomonas reinhardtii
Source: PLoS Biol. 2011 Jan 18;9(1):e1000577. doi: 10.1371/journal.pbio.1000577 (PMC3022525; doi:10.1371/journal.pbio.1000577)
Supplement: Table S1 — Picomoles of Zea, violaxanthin, and antheraxanthin before and after NPQ induction. De-epoxidation Index (Dep. Index) is calculated as (Z + A/2)/(Z + V + A). Data about A. thaliana from [102]. (0.02 MB PDF) [file pbio.1000577.s003.pdf]

Table S1: Picomoles of zeaxanthin, violaxanthin and antheraxanthin before and after NPQ induction. Dep. Index is calculated as (Z+A/2)/(Z+V+A). Data about *A.thaliana* from (1).

	Viola	Anthera	Zea	Chls	Dep. Index
**HL cells**	**6.7**	**1.0**	**1.0**	**100.0**	**0.2**
**HL cells after NPQ induction**	**3.8**	**1.7**	**1.9**	**100.0**	**0.4**
A. thaliana	**2.8**	**n.d.**	**n.d.**	**100.0**	**0.0**
***A. thaliana* after NPQ induction**	**1.4**	**0.4**	**2.3**	**100.0**	**0.6**

REFERENCES

1. Dall'Osto, L., S. Caffarri, and R. Bassi (2005) A mechanism of nonphotochemical energy dissipation, independent from PsbS, revealed by a conformational change in the antenna protein CP26. *Plant Cell* **17,** 1217-1232.
